# Strawberry Biostimulation: From Mechanisms of Action to Plant Growth and Fruit Quality

**DOI:** 10.3390/plants11243463

**Published:** 2022-12-10

**Authors:** Carlos Alberto Garza-Alonso, Emilio Olivares-Sáenz, Susana González-Morales, Marcelino Cabrera-De la Fuente, Antonio Juárez-Maldonado, José Antonio González-Fuentes, Gonzalo Tortella, Marin Virgilio Valdés-Caballero, Adalberto Benavides-Mendoza

**Affiliations:** 1 Protected Agriculture Ph.D. Program, Universidad Autónoma Agraria Antonio Narro, Saltillo 25315, Mexico; 2 Protected Agriculture Center, Faculty of Agronomy, Universidad Autónoma de Nuevo León, General Escobedo 66050, Mexico; 3 National Council of Science and Technology (CONACYT), Universidad Autónoma Agraria Antonio Narro, Saltillo 25315, Mexico; 4 Department of Horticulture, Universidad Autónoma Agraria Antonio Narro, Saltillo 25315, Mexico; 5 Department of Botany, Universidad Autónoma Agraria Antonio Narro, Saltillo 25315, Mexico; 6 Center of Excellence in Biotechnological Research Applied to the Environment, CIBAMA-BIOREN, Universidad de La Frontera, Temuco 4811230, Chile; 7 UPL Ltd. Mexico, Saltillo 25290, Mexico

**Keywords:** *Fragaria*, defense inducers, eustressors, elicitors, hormesis, plant stress, phytochemicals, nutraceutics, nutraceutical quality

## Abstract

The objective of this review is to present a compilation of the application of various biostimulants in strawberry plants. Strawberry cultivation is of great importance worldwide, and, there is currently no review on this topic in the literature. Plant biostimulation consists of using or applying physical, chemical, or biological stimuli that trigger a response—called induction or elicitation—with a positive effect on crop growth, development, and quality. Biostimulation provides tolerance to biotic and abiotic stress, and more absorption and accumulation of nutrients, favoring the metabolism of the plants. The strawberry is a highly appreciated fruit for its high organoleptic and nutraceutical qualities since it is rich in phenolic compounds, vitamins, and minerals, in addition to being a product with high commercial value. This review aims to present an overview of the information on using different biostimulation techniques in strawberries. The information obtained from publications from 2000–2022 is organized according to the biostimulant’s physical, chemical, or biological nature. The biochemical or physiological impact on plant productivity, yield, fruit quality, and postharvest life is described for each class of biostimulant. Information gaps are also pointed out, highlighting the topics in which more significant research effort is necessary.

## 1. Introduction

**Figure 1:**
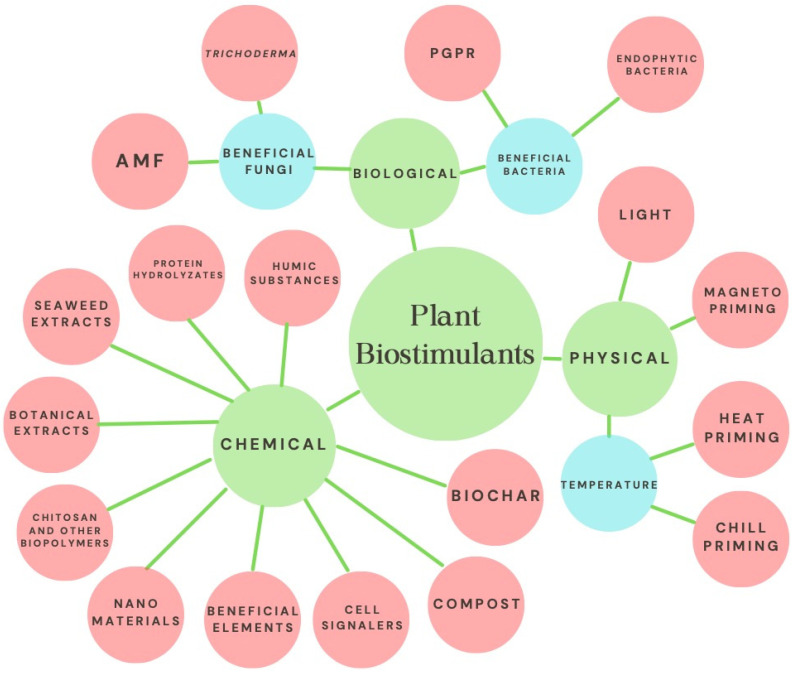
Main categories of biostimulants considered in this review. AMF: Arbuscular mycorrhizal fungi; PGPR: Plant growth-promoting rhizobacteria; Cell signalers: H_2_O_2_, H_2_S, NO. Figure prepared by the authors with information from various sources [4,5,6,7].

Biostimulation has gained relevance due to its positive effects on the growth and development of diverse crops. However, in the specific case of strawberries, there are currently no reports encompassing the various forms and techniques of application of biostimulants, as well as their mechanisms of action and positive effects on characteristics such as yield and nutraceutical quality of the fruits. In addition to the above, the constant increase in the population forces us to look for alternatives to achieve food security, since some projections estimate that food needs will be up to 70% higher by 2050 [1]. On the other hand, climate change has altered the conditions for agriculture, forcing growers to look for alternatives with new production systems and genotypes better adapted to increasing biotic and abiotic stresses [2]. The strawberry is a plant highly appreciated for its fruits of high organoleptic quality and significant commercial value; the worldwide harvested area exceeds 380,000 ha, with a production close to 9 million tons [3]. Plant biostimulation is a biological response that has been known empirically since ancient times, but its definition is recent. Plant biostimulation has been defined as applying any substance or microorganism to promote nutritional efficiency, tolerance to abiotic stress, and obtain higher quality crops, regardless of nutrient content [4]. Another definition refers to any material that can promote growth by being applied in small amounts to plants [5]. One of the most accepted categorizations includes the following groups of biostimulants: humic substances (humic and fulvic acids), protein hydrolysates, seaweed-botanical extracts, chitosan and other biopolymers, beneficial elements (Si, Se, I, Ti), beneficial fungi (arbuscular mycorrhizal fungi, Trichoderma) and beneficial bacteria (plant growth-promoting rhizobacteria and endophytic bacteria) [4]. However, other materials or stimuli that are not categorized in the above list can induce biostimulation in plants; these include compost, biochar, nanomaterials, as well as the exogenous application of signalers (H_2_O_2_, H_2_S, NO), and physical stimuli such as light (LED, UV), magnetism and high-low temperature (Figure 1).

The main ways biostimulants act in plants are through the active substances they contain, by having a large active surface or micro/nanoporosity, or through a complex system of recognition and signaling that is dependent on energy transduction or reducing potential. The aforementioned induces modifications in metabolism, membrane potential, membrane fluidity, and gene expression [6]. In addition, some groups of biostimulants (e.g., biopolymers, microorganisms, compost, and biochar) can act indirectly, mainly by modifying the physicochemical characteristics of the soil or substrate and promoting the assimilation of nutrients and the general growth of plants [7]. Some researchers have published reviews on applications of specific biostimulant categories in crops such as seaweed extracts [8]. However, to our knowledge, no review encompassing all forms of biostimulation in strawberry plants has been reported in the literature to date. Based on all the above, the objective of this work was to conduct a broad review of the literature related to the use of biostimulant products in strawberry cultivation, highlighting the impact of the forms and doses of application on the agronomic, physiological, and biochemical characteristics of strawberry plants. The literature search was carried out in the databases of Dimensions, Scopus, and Web of Science, considering publications from 2000–2022.

## 2. General Mechanism of Plant Biostimulation

### 2.1. Plant Cell Receptors

The first step in the process of biostimulation is the reception of stimuli from the environment. When any of the biostimulant agents (physical, chemical, biological) interacts with plant cells, the signal is perceived through various types of receptors or physiochemical changes in cell walls or membranes. The mechanisms of cellular reception to the stimulus perceived by biostimulants are not yet well known. However, they are likely related to the mechanism of perception of molecular damage by abiotic or biotic factors. The receptors are known as plant pattern-recognition receptors (PPRs) and are responsible for recognizing pathogen-associated molecular patterns (PAMPs) or damage-associated molecular patterns (DAMPs) [9]. One of the main groups of receptors is receptor-like cytoplasmic kinases (RLCKs), within which there are specific proteins that perceive different stimuli depending on their nature; one example is the chitin elicitor receptor kinase 1 (CERK1), which is responsible for the perception of chitin [10]. Another group of membrane receptors is the wall-associated kinases (WAKs), of which 26 genes related to Arabidopsis have been identified; these receptors perceive the stimuli to provide pathogen resistance, heavy-metal tolerance, and plant development [11]. Another critical group of receptors is the G Protein-Coupled Receptors (GPCRs), which perceive various types of extracellular stimuli and trigger signaling cascades to respond [12].

### 2.2. From Perception to Transduction and Signaling

**Figure 2:**
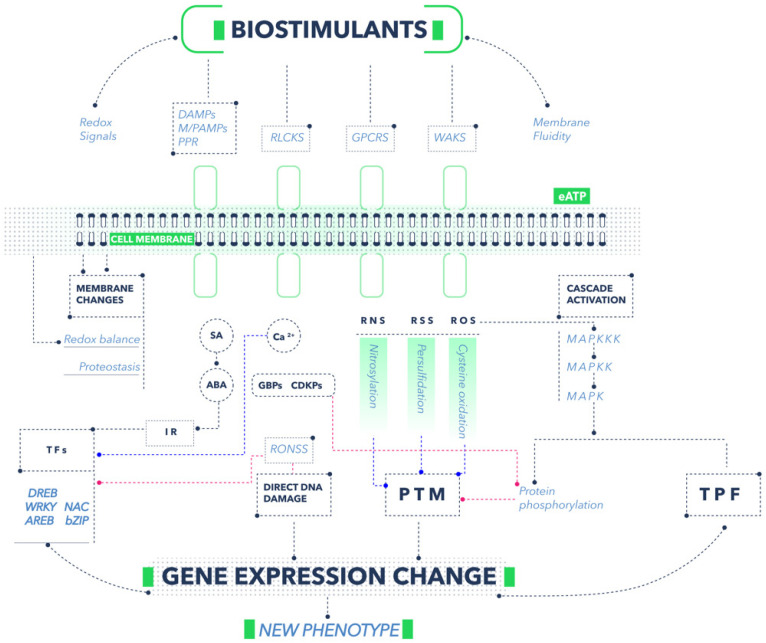
Mechanisms of action of biostimulants. The abbreviations used are defined in the text of this section. Figure prepared by the authors with information from various sources [9,10,11,12,13,14,15,16,17,18,19,20,21,22,23,24,25].

Once specific receptors perceive the stimulus, transduction of the signals immediately occurs, with various molecules or ions playing an important role [13]. Mitogen-activated protein kinases (MAPKs) are an example of proteins responsible for initiating a cascade of signaling that ranges from the perception of the stimulus to the arrival of information to other sites of the cell [6]. Usually, the process begins with the mitogen-activated protein kinase kinase kinases (MAPKKKs), following downstream toward the mitogen-activated protein kinase kinases (MAPKKs) and finally to the MAPKs. Protein phosphorylation is a type of posttranslational modification (PTM) [14] that alters proteostasis (protein homeostasis) in the cell medium. Proteostasis alteration is possibly recognized by cells and is partially responsible for inducing a biostimulation response in plants [15]. On the other hand, MAPKs can phosphorylate transcription factors that directly modify gene expression [6]. An essential element in signaling is Ca^2+^, which is a secondary messenger in plant cells. When the cell walls perceive a stimulus, the subsequent transduction response activates Ca^2+^ channels, and the cytoplasmic Ca^2+^ (Ca^2+^*cyt*) concentration increases. The change in Ca^2+^ is detected by various intracellular receptors, among which calmodulin (CaMs), calmodulin-like proteins, calcium-dependent protein kinases (CDPKs), and calcineurin B-like proteins stand out [16]. On the other hand, the high concentration of Ca^2+^*cyt* induces the production of Ca-binding proteins (CaBPs), modifying proteostasis in cells. Likewise, the increase in Ca^2+^*cyt* is fundamental for the phosphorylation of transcription factors by CDPKs [12]. Another compound that fulfills the role of a signaler is extracellular ATP (eATP), which is extruded from the cytoplasm to the apoplast when plants perceive some stimulus. This eATP is perceived by the membrane receptor called Does not Respond to Nucleotides 1 (DORN1), producing a response similar to that caused by DAMPs [17]. Some phytohormones, such as abscisic acid (ABA) and salicylic acid (SA), also play an important role in cell signaling. For example, when the membranes perceive some external stimulus, the cytoplasmic concentration of ABA increases, regulating genes related to resistance to salinity, drought, and cold stress [18]. Likewise, an elevation in the concentration of SA is detected by specific receptors, which favors the interaction with several transcription factors that modify the expression of genes mainly related to the defense system against biotic and abiotic stress [19]. On the other hand, when biostimulants first encounter cell walls and membranes, groups of important signalers arise. These signalers include reactive oxygen species (ROS), like H_2_O_2_, O_2_^−^, OH^−^; reactive nitrogen species (RNS), specifically NO and NO_2_; and reactive sulfur species (RNS), such as H_2_S, which can commonly be grouped together as reactive oxygen, hydrogen, and sulfur species (RONSS). One of the main biostimulation pathways is related to changes in the redox balance of cells when the RONSS:antioxidant ratio is increased in cells [20]. RONSS function as cell signalers due to their high reactivity and capacity to modify molecules by oxidation, nitrosation, nitration, or persulfidation. For example, ROS induce the oxidation of cysteine and methionine residues, which causes inactivation or changes in protein structures [21] (Figure 2). 

Additionally, there is evidence that some ROS are necessary to activate MAPK signaling cascades [6]. Likewise, NO fulfills various roles of PTM through mechanisms such as metal nitrosylation, tyrosine nitration, and S-nitrosylation [22]. On the other hand, H_2_S causes the persulfidation of proteins and residues such as cysteine, causing changes in the proteome and gene expression [23]. Gasotransmitters such as NO and H_2_S, thanks to their physical characteristics, can move quickly between organelles and through other cells, which increases their ability to induce transcriptional changes in plants [23]. In addition, all signalers are detected by other types of intracellular receptors and transcription factors, such as DREB, WRKY, AREB, NAC, and bZIP, thus modifying gene expression [24]. Signals can also travel directly to the nucleus of cells, causing changes in DNA and resulting in overexpression or repression of genes [25]. A final way in which plants respond to the stimuli of the environment is through changes in the fluidity and structure of membranes, which is like the observed effect when plants are subjected to stress due to salinity or drought [26]. Such changes in the membranes are perceived by putative sensors that subsequently modify gene expression [27]. Furthermore, some biostimulants have a large active surface per unit volume; examples are nanomaterials, zeolites, and biochar. The above materials can induce changes in plant behavior; this could be due to a physical interaction mechanism in the interphases of the material and cell walls, or related to the considerable ion-exchange capacity of the materials (see Section 3.7). The specific direct or indirect mechanisms by which the different categories of biostimulants positively affect the growth and development of plants, depending on their chemical, biochemical, biological, or physical nature, are described in the subsequent sections. As a result of all the previously mentioned mechanisms, a new phenotype better adapted to the environment is obtained, with greater tolerance to biotic and abiotic stress and better growth, development, and quality of harvestable products.

## 3. Use of Chemical and Biochemical Biostimulants in Strawberry Cropping

This group includes humic substances, protein hydrolysates, seaweed extracts, botanical extracts, chitosan and other biopolymers, beneficial elements, nanomaterials, compost, biochar, and cell signalers (H_2_O_2_, H_2_S, NO).

### 3.1. Humic Substances (HS)

Humic substances (HS) are organic compounds formed from plant or animal residues present in soils, which are degraded in a process known as humification resulting from the activity of microorganisms such as fungi and bacteria [28]. These substances represent approximately 25% of the total organic carbon present on the planet [29]. Depending on their characteristics, HS can be classified as humic acids (HA) and fulvic acids (FA), which differ mainly by their solubility, depending on the pH of the medium in which they are found [30]. The beneficial effects of HS on plants have been widely documented [31]. Part of the mechanisms of action is the ability to induce changes in the structure of the root system, promoting its growth and improving the assimilation of nutrients [32]. On the other hand, HS can act as antioxidant compounds, favoring some oxidation–reduction reactions in soils, substrates, or plant cells [33]. It is also likely that plants recognize the disordered molecular structure of HS, being detected as DAMPs and triggering a cascade of signals, as explained in previous paragraphs (See Section 2). Likewise, HS can improve soil structure, increase cation-exchange capacity, promote P solubility, and improve nitrate assimilation [34]. Therefore, in recent years, HS have been considered as plant biostimulants [35], with positive effects on plant growth and development. Different impacts of HS have been reported in the case of strawberry cultivation, which varies depending on the nature of the HS, dose, and forms of application of the products. The main positive effects reported include variables related to vegetative growth and yield, such as fruit quality, mineral concentration, and antioxidant compounds. However, there is very little, or no information related to metabolic aspects such as photosynthesis, and few studies related to the postharvest life of the fruit and tolerance to pathogens (Table 1).

### 3.2. Protein Hydrolysates (PHs)

Protein hydrolysates (PHs) are products that can be derived from animal origin (blood meal, leather byproducts, fish byproducts, and bird feathers) or vegetable origin (alfalfa hay, legume seeds, and other vegetables) [55]. Methods for producing PHs range from chemistry to thermal and enzymatic hydrolysis, depending on the source material [56]. The final content of free amino acids and other compounds will depend on the hydrolysis method, as some compounds are degraded during the process [57]. One of the main mechanisms of action of PHs depends on the high concentration of free amino acids and peptides, which function as signaling molecules, N sources, and metal-complexation or antioxidant metabolites [58]. The different peptides containing PHs can be recognized by plants through specific receptors, such as putative leucine-rich repeats (LRRs), triggering a cascade of signaling and transcriptional responses [56]. In addition to the above, some PHs also contain fatty acids, carbohydrates, phytohormones, and macro- and micronutrients, which fulfill their respective roles in plants [59]. On the other hand, PHs increase the activity of enzymes such as nitrate reductase (NR), nitrite reductase (NiR), and glutamine synthetase (GS); all of these are related to the assimilation of N in addition to promoting carbon metabolism, increasing the production of auxins and gibberellins, antioxidant enzymes, and photosynthetic pigments and secondary metabolites [55]. Furthermore, PH applications have been shown to stimulate flavonoid biosynthesis and the phenylpropanoid pathway [57]. Using PHs from various sources with various forms of application has shown positive effects on strawberry cultivation. In most cases, PHs are reported to increase variables related to vegetative growth and, to a lesser extent, to antioxidant compounds, chlorophylls, and minerals in tissues. However, information on aspects of primary metabolism and postharvest life of fruits is very scarce (Table 2).

### 3.3. Seaweed and Algal and Microalgal Extracts

Extracts of marine algae have gained importance in recent years due to the beneficial effects reported in various crops [67]. The main species used for producing these extracts are Ascophyllum nodosum, *Sargassum* spp., and *Laminaria* spp., among others [7]. The production of seaweed extracts is based on different methodologies, but mainly involve subjecting the biomass to high temperatures and pressures and using alkaline solutions to ensure the extraction of the active compounds [68]. An abundance of phenolic compounds, as well as the presence of phytohormones such as gibberellins, could be found within the specific mechanisms of action of seaweed extracts [69]. One of the main compounds found in these extracts is alginic acid, which can be perceived by plants and triggering a positive response; in addition, this substance favors the chelation of minerals in the soil, increasing the assimilation and accumulation of nutrients in plants [59]. In general, the positive effects of extracts on crop growth and quality are partially explained by the regulation of the genes RD29A, RD22, SOS, CBF3, COR15A, as well as the increase in osmolytes, greater efficiency in water use, and increase in photosynthetic pigments and mineral concentration [67]. Furthermore, these extracts improve the enzymatic and nonenzymatic systems of plants, providing greater tolerance to abiotic stress [70]. Seaweed extracts of several species with various forms of application have been reported in strawberry cultivation, highlighting some aspects of vegetative plant growth and fruit quality, mineral concentration, and enzymatic-nonenzymatic antioxidant systems. However, it is essential to have information related to transcriptomics and proteomics, resistance of plants to pathogens, and the postharvest life of fruits (Table 3).

### 3.4. Botanical Extracts

Botanical extracts are products generally derived from fresh plant tissues, especially from plants recognized for their high concentrations of bioactive compounds, minerals, phytohormones, and amino acids, among others [82,83]. Several species have been used to produce extracts; an example is the plant *Moringa oleifera*, of which there are several reports on its positive effects on plants [84,85]. However, despite all the above, the group of botanical extracts has not yet been sufficiently studied as a biostimulant because such products are mainly used as pesticides [4]. The methods for elaborating botanical extracts use solvents such as water or different alcohols, which are mixed with the biomass to be later stirred, blended, and even applied with ultrasound techniques [85]. The specific mechanism of action of botanical extracts is not yet well known. However, it is related to the high availability of minerals, amino acids, bioactive compounds, and phytohormones, which fulfill specific functions such as promoting growth and vegetative development, improving the antioxidant system, and greater tolerance to biotic and abiotic stress, among others [86]. Several works have been reported using botanical extracts as biostimulants in strawberry cultivation. An experiment in the open field with soil conditions and foliar applications of *M. oleifera* extract at concentrations of 2, 4, and 6% increased the fresh and dry weight of plants, the number of leaves, plant height, SPAD, carbohydrates, and the concentration of N, P, K, Ca, Mg Fe, Mn, and Cu, as well as some characteristics of fruits, such as weight, firmness, TSS, Vit. C, anthocyanins, and total yield [87]. On the other hand, foliar applications of a mixture of three grass species, *Lolium perenne* L. (60%), *Festuca* spp. (20%), and *Poa pratensis* L. (20%) promote root and shoot dry weight and chlorophyll concentration in strawberry plants grown under greenhouse conditions [88]. In a similar experiment carried out using the same botanical extract in the strawberry plant cv. Diamond, foliar applications increased shoot and root dry weight, chlorophyll, and concentrations of succinic, malic, and citric acid in root tips, as well as concentrations of P, K, Mg and Ca in different organs of the plant [89]. On the other hand, drench applications of a *Pelargonium hortorum* extract increased some parameters of the radicular system, such as root diameter and root volume, as well as the photosynthetic rate in strawberry plants cv. Duch [61]. 

### 3.5. Chitosan and Other Biopolymers

Biopolymers are compounds widely used in the pharmaceutical, cosmetic, textile, and food industries. The main ones are cellulose, collagen, alginate, chitin, and chitosan, which have the most significant applications in agriculture [90]. Chitosan is a biopolymer obtained through the chemical or enzymatic deacetylation of chitin, mainly from crustaceans or insects, where the result can be D-glucosamine and N-acetyl-D-glucosamine [91]. Deacetylation consists of replacing acetyl groups (CH_3_CO) with amino groups (NH_2_), where the degree of this process (reaction time and temperature) defines the final form of chitosan (D-glucosamine or N-acetyl-D-glucosamine) [91]. The multiple applications of chitosan are due to its biocompatibility, biodegradability, high absorption capacity, and nontoxicity [92]. In plants, chitosan is mainly used to improve the response against pathogens and resistance to abiotic factors, in addition to promoting vegetative growth [90]. The primary mechanism of action of chitosan applications could be related to the octadecanoid pathway, which begins in the chloroplast of the cell and ends in the production of response genes related to enzymes such as PAL and CAT, as well as other response mechanisms such as stomatal opening/closing [93]. Signals ranging from chitosan perception to transduction factors include NO, Ca^2+^, and phytohormones such as JA, SA, and ABA [94]. Currently, no specific receptors have been identified for chitosan. However, the first perception could be related to the difference in charges between the amino groups of chitosan (positive charge) and the cell membrane (negative charge) [93]. The forms of chitosan application in plants range from seed priming, drench, and leaf sprays, while beneficial effects range from increased biomass gain, more photosynthetic pigments, and antioxidant compounds [95]. Some reports of the application of this product in strawberry cultivation are shown in Table 4. In this Table, the emphasis is placed on aspects related to fruit quality (size, weight, TSS, firmness, yield), postharvest life, antioxidant system, and, to a lesser extent, the concentration of minerals. There is little or no information related to the physiological issues of plants.

### 3.6. Beneficial Elements

Beneficial elements are not considered essential for plants, but their presence or application positively affects growth and development parameters [104]. The most studied elements in this group are silicon (Si), selenium (Se), iodine (I), vanadium (V), cobalt (Co) and titanium (Ti) [105]. These elements can be considered biostimulants because they can promote plant growth and provide tolerance to stress through mechanisms such as strengthening cell walls, osmoregulation, synthesis of phytohormones, greater assimilation of essential elements, and reduction of transpiration, among others [4]. Si is the most beneficial element studied; several authors have considered it a biostimulant for plants [106]. Among the main functions of Si in plants is its ability to accumulate in cell walls, providing greater rigidity to tissues and reducing damage by organisms such as insects or microorganisms [107]. In addition, Si can reduce the absorption of ions such as Na^+^ and Cl^−^ when plants are under saline stress conditions [108] and increase the production of antioxidant compounds in the face of various types of biotic and abiotic stress [106]. On the other hand, Se promotes the quenching of ROS, regulates enzymatic and nonenzymatic antioxidants, and improves the photosynthesis and homeostasis of elements in plants [109]. Likewise, iodine has been an element of interest in recent years, where its functions are mostly related to the increase in antioxidant compounds when this element is at low concentrations; however, high concentrations produce phytotoxicity in cells [110]. Finally, V, Co, and Ti are the elements less studied. However, it has been reported that these elements promote the assimilation of other nutrients, are involved in redox reactions, and stimulate enzymatic activity and photosynthesis [104,105,111]. These elements have been applied in strawberry cultivation, obtaining favorable responses in various groups of variables, such as agronomic (growth and development), fruit quality (size, weight, firmness, TSS, anthocyanins), the antioxidant system of the plant, aspects related to photosynthesis (photosynthetic rate, stomatal conductance), and the concentration of minerals in the tissues. However, further studies related to the tolerance against pathogens and postharvest quality of the fruits are needed (Table 5).

### 3.7. Metal, Carbon, Zeolite, and Chitosan Nanomaterials

Nanotechnology has gained importance in recent years due to its applications in industry, medicine, and agriculture, with uses such as pesticides or fertilizers found in the latter [140]. Nanomaterials (NMs) are considered products of a size between 1–100 nm, ranging from metals (ZnO, FeO_3_, SiO), carbon (carbon and graphene nanotubes), zeolite, and nanochitosan [141]. Recently, nanomaterials (NMs) have been proposed as plant biostimulants [5]. The positive effects of NMs in plants can be explained by the specific mechanisms by which NMs induce biostimulation in plants, which can be encompassed in two main phases: The first phase is due to the initial contact of the material with the cell walls or membranes, where interactions occur due to the difference in corona composition, surface charges, size, shape, and hydrophobicity of the NMs. NMs cause damage or modifications in the structures of integral proteins, cell walls, or membranes. These, in turn, can produce cascades of signalers (signaling metabolites, alterations of the redox balance, the membrane potential, and transcriptional and posttranslational modifications) inside or between cells and trigger a biostimulation response [5,142]. Once NMs cross the cell membrane through existing pores, inducing new pores or mechanisms such as diffusion or endocytosis, a series of similar reactions usually occur between NMs and organelles such as the nucleus, mitochondria, or chloroplasts [143]. In the second phase, once the NMs are internalized and transported through plant cells, the biotransformation of the NM core into specific ions (e.g., Zn, Fe, Cu, Si) occurs. The ions will be available in the cytoplasm of the cells and can fulfill specific roles in the metabolism of plants [144]. Several reports of NM applications in strawberry plants can be found in Table 6, where greater interest has been placed on the effects on vegetative growth, quality of fruits, bioactive compounds, and, to a lesser extent, the concentration of minerals and organic acids in tissues. There is little information regarding the biotic stresses and the postharvest life of the fruits.

### 3.8. Compost

The decomposition of organic matter forms composts with the help of soil microorganisms. The primary sources of organic matter come from plant wastes or manure of animal species used in livestock such as birds, cows, pigs, and horses [163]. In addition to the conventional form of composting, it is possible to use worms to obtain a product known as vermicompost [164]. Although some authors do not consider compost as a biostimulant [4], the applications of these products to soil or any other culture medium have shown some of the beneficial effects shown by other types of biostimulants [165]. Due to the limited study of this category as a biostimulant, the mechanisms of action are also unknown. However, most of them are related to indirect mechanisms, such as the increase in the populations of beneficial microorganisms, buffer for electrons and protons in the soil volume, increased moisture retention, and increased fertility, among others [165,166]. The composts contain a high concentration of humic substances that fulfill the roles previously explained (see Section 3.1), in addition to having high amounts of beneficial fungi and bacteria with biostimulant potential (see Section 4). Although the primary way of applying compost is directly as a mixture with the soil or substrates, it is also possible to elaborate extracts known as “compost tea”, which can be applied in a drench or foliar [167]. Composts from various sources have been used at different levels and forms in strawberry cultivation (Table 7). Most studies report beneficial effects on vegetative growth, yield, quality of fruits, and the concentration of minerals in leaves and fruits. However, there is a lack of information on variables such as photosynthesis, antioxidant compounds, postharvest quality of fruits, and resistance of plants to pathogens.

### 3.9. Biochar

Biochar, also called biocarbon or vegetable carbon, is a product obtained from transforming organic matter with high temperatures and the absence of oxygen, a process known as pyrolysis [183]. The composition and physicochemical characteristics vary depending on the organic matter origin and the pyrolysis temperature. Biochar is a compound with a porosity up to 124 m^2^ g^−1^ [184], rich in N, and with high concentrations of humic substances [185]. Like compost, biochar is not commonly studied as a biostimulant; however, some of its effects on soil characteristics promote plant growth, development, and quality [186]. Among the indirect mechanisms by which biochar could be considered a biostimulant are its abilities to improve soil structure by increasing porosity that facilitates the movement of air, water, and nutrients in the soil [187]. In addition to the afore-mentioned effects, biochar can increase soil pH, promote cation-exchange capacity, and increase efficiency in using N, among others [166]. The application of biochar to the soil favors root colonization and the activity of plant growth-promoting rhizobacteria (PGPR) [188]. One of the main effects of biochar applications in strawberry cultivation is the capacity to reduce the incidence of diseases in leaves and fruits. A study reported that wood-biochar and greenhouse-waste biochar (mixed with soil at 1–3%) mediate the systemic response of strawberry plants against *Botrytis cinerea*, *Colletotrichum acutatum*, and *Podosphaera apahanis,* promoting the overexpression of defense genes such as *FaPR1*, *Faolp2*, *Falox*, and *FaWRKY1* [189]. On the other hand, a recent investigation reported that biochar application mixed with peat substrate had a positive effect on the resistance of strawberry fruits against *Botrytis cinerea*, which was attributed to changes in the microbial community of the substrate [190]. Biochar application (1% in peat substrate) promotes fresh and dry weight and a lower susceptibility to the fungal pathogen *Botrytis cinerea* on both leaves and fruits of strawberry plants [191]. On the other hand, animal-bone biochar (130 kg ha^−1^) and plant-based biochar (1 ton ha^−1^) improve the number of fruits and total yield of strawberries grown in soil under open field conditions [192].

### 3.10. H_2_O_2_, NO, H_2_S, H_2_, CH_4_, and CO

Cell signalers play a key role in the biostimulant response of plants, as explained in Section 2.2. In recent years, the exogenous application of these compounds has been studied due to the positive effects observed in various plant species [193]. In some cases, it is possible to directly apply the molecule of interest (such as H_2_O_2_); however, in the case of gasotransmitters, precursor compounds must be used, such as sodium nitroprusside (SNP; source of NO) and NaHS (source of H_2_S) [194]. All these compounds are applied in very low doses since high concentrations could cause damage to plants. The primary responses are related to the increase in the activity of antioxidant enzymes and the production of nonenzymatic antioxidant compounds to maintain redox balance [195]. Some exogenous applications of signalers have been reported in strawberry plants, with greater emphasis given to H_2_O_2_, NO, and H_2_S and the response of enzymatic and nonenzymatic antioxidant compounds, vegetative growth, and fruit quality (Table 8).

## 4. Use of Biological Biostimulants in Strawberry Cropping

Biological biostimulants, also known as biopreparations or bioformulations, are products characterized as containing some living organisms, usually microorganisms such as bacteria and fungi, as the main active ingredient [208]. In the group of bacteria, we found plant growth-promoting rhizobacteria (PGPR) and endophytic bacteria, while in the group of fungi, we found arbuscular mycorrhizal fungi (AMF) and fungi of the genus *Trichoderma*. The main characteristics of each group, as well as its applications in strawberry cultivation, are described below.

### 4.1. Beneficial Bacteria

#### 4.1.1. PGPR

The group of plant growth-promoting rhizobacteria (PGPR) includes multiple species, where the genera *Bacillus*, *Pseudomonas*, *Azospirillum*, *Rhizobium*, and *Streptomyces* stand out [209]. In the market, it is possible to find commercial formulations with one or several species of bacteria combined, where applications have shown positive effects on crop growth and development [210]. The mechanisms of action of PGPR in plants can be direct or indirect. Among the direct mechanisms are the production of phytohormones such as auxins, indole acetic acid, gibberellins, and cytokinins, which regulate the growth and development of plants [7]. Additionally, some species of PGPR can produce volatile compounds that promote plant growth [211] in addition to increasing tolerance to various types of stress through the induction of the production of antioxidant enzymes in plants, modulation of membrane integrity, and accumulation of osmolytes [188]. In contrast, indirect mechanisms are the biological fixation of N, solubilization of P and other elements in soils, and production of metabolites, among others [212]. For products containing soil-colonizing bacteria, the application forms must be carried out directly to the root zone, either in drench, direct mixing with the soil or substrate, or root dipping, before transplanting to the final place [211]. Several reports of PGPR applications in strawberry plants can be found in Table 9, where a wide diversity of agronomic variables, yield and quality of fruits, antioxidant system, concentration of minerals, and, in some cases, variables related to photosynthesis have been studied. However, studies related to biotic and abiotic stresses and postharvest are necessary.

#### 4.1.2. Endophytic Bacteria

Endophytic bacteria are characterized by colonizing the internal tissues of plants and crossing the root epidermis to reach the vascular bundles, through which they can reach the stems, leaves, flowers, and fruits [210]. Most endophytic species include *Bacillus, Pseudomonas*, *Azospirillum*, *Rhizobium*, and *Streptomyces* [209]. The mechanisms of action of this group of microorganisms are like those mentioned in the section PGPR, to which are added: the increase of cellulose, providing greater resistance to the attack of herbivores; reduction of toxicity by heavy metals through extracellular precipitation, sequestration or biotransformation; and modifications in gene expression to increase defense by pathogens [231]. On the other hand, one of the main characteristics of endophytic bacteria is the production of siderophores, which function as chelating agents of Fe, promoting the assimilation of this element by the roots [232]. Several reports of endophytic bacteria use in strawberry plants can be found in Table 9; however, unlike the PGPR group, only effects have been reported on variables related to vegetative growth and some antioxidant compounds.

### 4.2. Beneficial Fungi

#### 4.2.1. Arbuscular Mycorrhizal Fungi (AMF)

Arbuscular mycorrhizal fungi (AMF) are different species of fungi characterized by a symbiotic association with plant roots [233]. The main species of AMF are *Rhizophagus intraradices* (formerly known as *Glomus intraradices*), *Funneliformis mosseae* (formerly known as *Glomus mosseae*), and some species of the genus *Gigaspora* [234]. One of the main characteristics that identify AMF is the ability to form an extension of up to 40 times the root system of plants, exploring a greater volume of soil [233]. This functional root surface expansion explains the main mechanisms of action by which AMF are considered biostimulants, since they allow an increase in the absorption of water and nutrients, produce P solubilizing compounds in the soil, alter the architecture of the root, produce antioxidant compounds and induce signaling phytohormones such as ABA [59]. In addition, AMF provide plants with greater resistance to abiotic stress—such as drought, salinity, nutritional deficiencies, heavy metals, and changes in pH—due to the production of ascorbic acid, phenolic compounds, flavonoids, and carotenoids when the roots perceive the stimulus caused by AMF [234]. Several reports of AMF applications in strawberry plants can be found in Table 10. Most of the studies focus on determining the mineral concentrations in tissues, vegetative growth, and the antioxidant system of plants, with some related to photosynthetic variables. However, in this category, reports on the effects of AMF on fruit quality and postharvest life are lacking.

#### 4.2.2. Trichoderma

*Trichoderma* is a genus of beneficial fungi for plants that comprise more than 200 species; *Trichoderma harzianum* is the most studied [253]. These fungi are characterized by their usual endophytic growth habit, penetrating through the roots of plants [254]. Therefore, plants perceive the stimulus by the spores or mycelia of the fungus, obtaining a response similar to the microorganisms described in the previous Section 4.1.1, Section 4.1.2 and Section 4.2.1. Among the primary mechanisms of action of *Trichoderma* is the modulation of hormonal signaling by ABA, ET, JA, and IAA, in addition to favoring the activity of MAPK cascades [253]. On the other hand, inoculation with *Trichoderma* increases the assimilation of elements such as P, Mg, Zn, Fe, and B [55]. There are also reports where the absorption and efficiency in using N were increased [254]. On the other hand, *Trichoderma* can produce antioxidant compounds such as glucosinolates and phytoalexins, which allow counteracting the attack of other phytopathogenic microorganisms [255]. Additionally, some reports indicate that *Trichoderma* increases the populations of some beneficial bacteria in soils [256]. Colonization with *Trichoderma* also induces changes in the plant proteome, modifying the synthesis of proteins involved in essential processes such as carbohydrate metabolism and photosynthesis, among others [253]. Several reports of *Trichoderma* inoculation in strawberry plants can be found in Table 10. Although there are few reports on the application of this microorganism in strawberry plants, research has covered aspects related to vegetative growth, fruit quality, and photosynthetic variables. However, more research is needed regarding the strawberry antioxidant system and tolerance to pathogens.

## 5. Use of Physical Biostimulants in Strawberry Cropping

This group includes supplementary applications of light (mainly through LEDs), priming with extreme temperatures (high or low) and treatments with magnetism.

### 5.1. Biostimulation and Priming Using UV and Visible Light

Supplementation with artificial light, either visible or UV light, has been shown to have positive effects on plant growth and development [257]. In the first instance, visible light supplementation, mainly within the photosynthetically active radiation range (PAR: 400–700 nm), increases the photosynthetic activity of plants [258], resulting in more significant dry matter gain and crop yields. However, another mechanism is the ability to stimulate plants, induce morphological and anatomical changes, and regulate some developmental processes, such as flowering [259]. Plants have specific receptors for different wavelengths, including phytochromes (red/far red light, 600–750 nm), cryptochromes (blue, 350–500 nm), phototropins, F-box-containing flavin-binding proteins (blue/UV-A, 320–500 nm), and UVR8 (UV-B, 280–320 nm) [260]. Once these receptors perceive a light stimulus, signal transduction is carried out mainly through ROS [261] and hormonal signalers such as IAA, brassinosteroids, and ethylene [262,263]. Once TFs detect the signals, the changes in gene expression are like those reported for other groups of biostimulants. Some studies have shown the positive effects of different types of supplementary light on strawberry cultivation (Table 11). Due to the nature of this biostimulant method, most research has focused on studying some photosynthetic parameters (e.g., stomatal conductance, CO_2_ assimilation, photosynthetic rate), as well as vegetative growth and fruit quality. Information on antioxidant compounds, pathogen resistance and postharvest life of fruits is still scarce.

### 5.2. Biostimulation and Priming Using Heat Shock and Chill Priming

Plants have various mechanisms to respond to temperature changes in the air or rhizosphere. This category of biostimulation consists of subjecting plants for a certain time to high or low temperatures, without them becoming lethal, which triggers a response to achieve acclimatization. Some of the thermo-sensors identified in plants are glutamate receptor-like (GKR) and cyclic nucleotide-gated channels (CNGCs) [273]; however, plants also use some of their photoreceptors, such as phytochromes and phototropins, to perceive stimuli by temperature [274] and begin the transduction of signals, mainly through signaling by Ca^2+^*cyt*, H_2_O_2_, and NO [275]. These signalers reach the heat shock transcription factors (HSFs), which have been identified as at least 20 members, from which the overexpression of the *HSP90* and *HSP70* genes occurs [276]. These genes produce heat shock proteins (HSPs), which are proteins that reduce molecular damage caused by temperature extremes [277]. In an experiment carried out in strawberry fruits subjected to a temperature of 45 °C for 3.5 h, an increment was found in the activity of the enzymes chitinase (CHI), β-1,3-glucanase, PAL, SOD, CAT, and APX, providing resistance against the fungus *B. cinerea* [278]. In addition, Widiastuti et al. [279] performed root dipping of strawberry seedlings in water at different temperatures (40, 45, and 50 °C) for 20 s, as well as immersion of the basal leaf in water at 50 °C for 20 s. In both cases, they found overexpression of the *CHI2-1* gene, the precursor of the CHI enzyme. They also reported an increase in the concentration of salicylic acid (SA) in leaves. All the above resulted in a decrease in the incidence of the fungus *Colletotrichum gloeosporioides*, which causes strawberry crown rots. In another work carried out by Brown et al. [280], strawberry roots were placed in a water bath at 37 °C for 1 h, resulting in the overexpression of genes related to the synthesis of heat shock proteins (HSP), such as *HSP90* and *HSP70*, which would mean a greater tolerance to heat shock stress in strawberry plants. Kesici et al. [281] placed strawberry plants in growth chambers under different high-temperature treatments (35, 40, 45, and 50 °C) for 24 h and also found overexpression of the *HSP90*, *HSP70*, and small heat shock protein (sHSPS) genes, seen as an increase in soluble protein in plants.

### 5.3. Magnetopriming

Magnetopriming consists of subjecting seeds or other plant organs to a magnetic field for a specific time to produce changes in metabolism [282]. The mechanisms by which magnetic fields act in plants are not yet well known. However, it is most likely that they are related to changes in the electrical charges of cellular components, producing reorganizations of the various structures [283]. Likewise, magnetopriming increases the production of ROS such as H_2_O_2_ and O_2_^−^ [284], favoring signaling cascades in plants. On the other hand, it has been reported that magnetism induces the production of enzymatic and nonenzymatic antioxidant compounds, providing greater tolerance to different abiotic stresses, such as saline stress [285]. Therefore, magnetopriming can be considered a form of biostimulation since numerous works have reported positive effects on plants, such as more significant vegetative growth, increased photosynthesis, and favoring germination, among others [286]. Currently, there are no reports on the use of magnetism for the biostimulation of strawberry plants.

**Figure 3:**
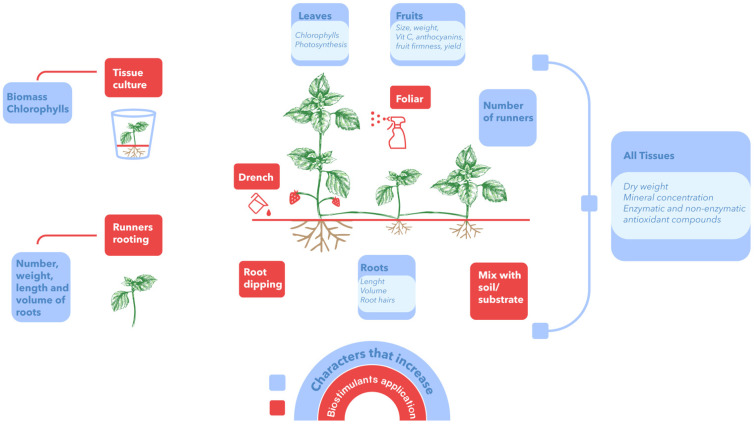
Forms of application of biostimulants and main effects on strawberry plants. Figure prepared by the authors with information reported in the tables of this review.

As a general summary, Figure 3 presents the main ways of applying biostimulants in strawberry plants, as well as the parameters of interest that are increased in this crop.

## 6. Comments and Future Perspectives

The application of biostimulant products in strawberry cultivation has constantly been evolving over the years. However, as seen in this review, for some of the categories of biostimulants, there are still few reports on their effects on this crop, which can be explained due to their more recent discovery or development, as is the case for the categories of nanomaterials or magnetopriming. In contrast, biostimulants types such as humic substances, protein hydrolysates, and composts have more reports in the literature, most of them in the years prior to 2010. For beneficial microorganisms, this review presents reports since 2000. However, their biostimulant potential has been known for a long time and is still a source of new information derived from research and field applications. As previously mentioned, new categories of biostimulants such as nanomaterials, beneficial elements, and physical methods (temperature, light, magnetism) have become very important in recent years. Therefore, in addition to studying the positive effects on the growth and development of plants, there is also interest in explaining the physiological, biochemical, and metabolic mechanisms by which these biostimulants produce responses in plants. In addition to the categories considered in this review, it is possible that, in coming years, new definitions and classifications of biostimulants will emerge. Thus, the constant evaluation of new physical, chemical, and biological agents is of utmost importance, not only to focus on characteristics of agronomic interest, but also to pay greater interest to the mechanisms of action of the biostimulants applied to plants; this in turn will allow us to develop new techniques to increase the nutraceutical quality of strawberries, add to a higher fruit yield and increase resistance to biotic and abiotic stress factors.

## 7. Conclusions

The reviewed reports indicate that the great variety of biostimulants and ways of applying them exert a beneficial effect on the plant’s agronomic, physiological, and biochemical variables, with an equally favorable impact on the quality variables of the strawberry fruit. Regarding the variables mentioned above, those related to vegetative growth and fruit quality have received more significant interest. Nevertheless, it is necessary to study in-depth responses in the antioxidant system of plants and some physiological variables, such as photosynthesis, in addition to some studies referring to the postharvest quality of strawberries. Although most categories of biostimulants have been studied for physiological, biochemical, and molecular mechanisms, in some categories (e.g., gasotransmitters, botanical extracts, compost, biochar, nanomaterials, and physical biostimulants), the plant responses are poorly understood. As a result, there are great opportunities to conduct research in different biostimulation areas that have not yet been sufficiently explored in strawberries.

## Figures and Tables

**Table 1:** Positive effects of HS on some growth or quality variables of strawberry crops.

Product	Experimental Conditions	Forms and Levels of Application	Variables That Increase	Reference
HA NS *	Greenhouse, pots with substrate	Foliar 0, 25, 50, and 100 mg L^−1^	Fruit yield, TSS, TA, Vit. C, K, P, Ca, Mg.	[36]
HA from cow manure, food waste, paper waste	Greenhouse, pots with substrate	Substrate mix 0, 250, and 500 mg kg^−1^ of substrate	Root dry weight.	[37]
HA from cow manure, food waste, paper waste	Greenhouse, pots with substrate	Substrate mix 0, 250, and 500 mg kg^−1^ of substrate	Number of fruits.	[38]
HA Commercial formulation	Open field, pots with soil	Root immersion by 2 h, 0.05%	Number and length of runners, length of roots, and total biomass.	[39]
HA + FA Commercial formulation	Greenhouse, pots with substrate	Substrate mix, 0.06 g kg^−1^	P in roots, Mn and P in leaves.	[40]
HA NS	Greenhouse, pots with soil	Foliar 15 and 25 mL L^−1^	Biomass, length of roots, leaf area, number of runners and flowers, fruit weight, TSS, TA, and Vit. C.	[41]
HA NS	Open field, soil	Foliar 0, 2, and, 4 mL L^−1^	N concentration in leaves, number of flowers, and fruit yield.	[42]
HA NS	Greenhouse, pots with soil	Foliar 100 mg L^−1^	Proline concentration, phenolics, and antioxidant capacity.	[43]
HA Commercial formulation	Greenhouse, pots with substrate	Substrate mix 4 g HA pot^−1^	Plant height, number of leaves, crowns, and roots, fresh and dry weight of leaves and roots, stomatal conductance.	[44]
HA + FA Extracted from vermicompost	Open field, soil conditions	Foliar 180 mg L^−1^	Chlorophyll concentration and net photosynthesis.	[45]
HA NS	Greenhouse, soil conditions	Foliar 20 and 40 mg L^−1^	Number and weight of fruits, yield per plant, leaf area, length and dry weight of shoot and root.	[46]
HA + FA NS	Open field, soil conditions	Drench 5 mL L^−1^	TSS, TA, anthocyanins, Vit. C, phenolics.	[47]
HA + FA Commercial formulation	Open field, soil conditions	Drench and Foliar 2, 4, and, 6 ton ha^−1^	Leaf area, biomass, chlorophyll, carotenoids, TSS, and Vit. C.	[48]
HA Extracted from soil	In vitro	Growing medium 1 and 5 mg dm^−3^	Number and length of roots, plant weight, number and size of leaves.	[49]
HA Commercial formulation	Greenhouse, pots with substrate	Drench 150 and 300 mg L^−1^	K concentration, chlorophyll, carbohydrates, shoot and root dry weight, leaf area, SOD, fruit number and yield.	[50]
HA Commercial formulation	Greenhouse, pots with substrate	Foliar 1 g L^−1^	Root dry weight, Si, fruit chromaticity.	[51]
HA NS	Greenhouse, soil conditions	Drench and foliar 10, 20, 30, and 40 mg L^−1^	Chlorophyll, N, P, K.	[52]
HA NS	Greenhouse, pots with soil	2 g kg^−1^ soil	Plant height, leaf area, fresh weight, N, P, K.	[53]
HA + FA Commercial formulation	Open field, soil conditions	Drench 10 mL L^−1^	Number and length of runners; number, length, and weight of roots.	[54]

* NS: Not Specified.

**Table 2:** Positive effects of protein hydrolysates on some growth or quality variables of strawberry crop.

Product	Experimental Conditions	Forms and Levels of Application	Variables That Increase	Reference
Porcine blood	Open field, soil conditions	Drench 0.5, 1, and 1.5 g plant^−1^	Resistance to cold stress, fruit weight.	[60]
Fish protein concentrates	Greenhouse, pots with soil	Drench NS	Fresh and dry biomass, chlorophyll fluorescence.	[61]
Amino acids (Proline, Alanine, Glutamine	In vitro	Growing medium 50, 100, 150, and 200 mg L^−1^	Somatic embryogenesis.	[62]
Porcine blood	High-tunnel, soil conditions	Drench 2.5 g L^−1^	Dry weight of roots, % of flowering, fruit weight.	[63]
Arginine NS	Greenhouse, soil conditions	Foliar 0, 250 and 500 μM	Number of fruits, TSS, anthocyanins, phenolics, Vit. C.	[64]
Alfalfa protein hydrolizated	Greenhouse, pots with substrate	Foliar 3 g L^−1^	Root dry weight, leaf area, Si concentration, SPAD, fruit weight, phenolics.	[51]
Microalga protein hydrolizated	Greenhouse, pots with substrate	Foliar 4 g L^−1^	Root dry weight, Fe and Si concentration in roots, TA in fruits.	
Mix of amino acids	Greenhouse, pots with substrate	Foliar 3 g L^−1^	TSS in fruits.	
Amino acids (hydroxyproline and glutamic acid), commercial formulation	Controlled environment room, pots with substrate	Foliar 228 and 319 mg L^−1^	Number of flowers, number, and weight of fruits, Vit. C.	[65]
Hydrolyzed feather meal	Greenhouse, pots with soil	0.10 g kg^−1^ soil	Indole Acetic Acid (IAA), Abscisic acid (ABA), Isopentenyl adenosine (iPA).	[66]
Amino acids (Glycine)	Open field, soil conditions	Drench 0.5 g L^−1^	Number and length of runners, roots length.	[54]

**Table 3:** Positive effects of seaweed and microalgal extracts on some growth or quality variables of strawberry crops.

Product	Experimental Conditions	Forms and Levels of Application	Variables That Increase	Reference
*Ascophyllum nodosum*, commercial extract	Greenhouse, pots with substrate	Drench 0.2, 0.4, 1.0, or 2.0 g L^−1^	Number, surface area, volume, and length of roots.	[71]
	Open field, soil conditions	Drench 2 and 4 g L^−1^	Leaf area, shoot dry weight, number of fruits and yield.	
*Sargassum* spp., commercial extract	Open field, pots with substrate	Drench 0, 2, 4, and 8 g L^−1^	Mn concentration.	[72]
*Sargassum* spp., commercial extract	Open field, pots with substrate	Drench 0, 2, 4, and 8 g L^−1^	Number of crowns, number and volume of fruits, yield.	[73]
*Ascophyllum nodosum*, commercial extract	Greenhouse, pots with substrate	Foliar 0.1, 0.2, and 0.3%	Phenolics and flavonoids concentration; activity of PAL and POD. More resistance to *Podosphaera aphanis*.	[74]
Seaweed extract, NS	High tunnel, soil conditions	Drench 20 g ha^−1^	Concentration of N, P, K, Ca, Mg, and Mn.	[75]
Mix of *Sargassum* sp., *Ascophyllum* *nodosum*, *Laminaria* sp.	Open field, soil conditions	Foliar 1 and 2 mL L^−1^	Plant height, number of leaves, leaf area, root dry weight, fruit weight, TSS.	[76]
*Ascophyllum* *nodosum*, commercial extract	Open field, soil conditions	4.68 L ha^−1^	Number of crowns, root dry weight, fruit yield.	[77]
Seaweed extract, NS	High tunnel, soil conditions	Foliar 1.3 g L^−1^	Leaf area, fruit N concentration, fruit yield.	[78]
Seaweed extract, NS	High tunnel, soil conditions	Foliar 1.3 g L^−1^	TSS, fructose, sucrose, and quercetin.	[79]
Mix of *Duvillaea potatorum* and *Ascophyllum nodosum*	Open field, soil conditions	10 L ha^−1^	Number of runners, fruit yield, roots length.	[80]
Seaweed extract, NS	Open field, soil	Foliar 2 and 4 mL L^−1^	Leaf and root dry weight, N concentration, number of flowers, yield.	[42]
*Ascophyllum* *nodosum*, commercial formulation	Greenhouse, pots with substrate	Foliar 3 g L^−1^	Root dry weight, leaf area, Si in roots, phenolics.	[51]
*Spirulina* spp., commercial formulation	Greenhouse, pots with substrate	Foliar 3 g L^−1^	Root dry weight, Fe and Si in roots, fruit firmness and TA.	
*Ascophyllum* *nodosum*, commercial formulation	Greenhouse, pots with substrate	Drench 0.5 mL L^−1^	Vegetative growth, chlorophyll concentration, photosynthetic rate, number, and weight of fruits.	[81]

**Table 4:** Favorable effects of chitosan applications on some growth or quality variables of strawberry crop.

Product	Experimental Conditions	Forms and Levels of Application	Variables that Increase	Reference
Chitosan, commercial product	Open field, soil conditions	Foliar 1, 2, 3, and 4 mL L^−1^	Plant height, number of leaves, biomass, number and weight of fruits.	[96]
Chitosan, commercial product	Open field, soil conditions	Foliar 125, 250, 500, and 1000 mg L^−1^	Leaf size, fresh and dry weight of shoot and roots, fruit weight and yield.	[97]
Chitosan, commercial product	Open field, soil conditions	Foliar 125, 250, 500, and 1000 mg L^−1^	Anthocyanins, phenolics, flavonoids, carotenoids, antioxidant capacity.	[98]
Chitosan oligosaccharide, commercial formulation	Open field, soil conditions	Foliar 50 mg L^−1^	Fruit firmness, TSS, Vit. C, phenolics, flavonoids, antioxidant capacity.	[99]
Chitosan, commercial product	Greenhouse, pots with substrate	Foliar 10 mL L^−1^	Root dry weight, B and Si concentration in roots, weight, firmness, and fruit yield.	[51]
Chitosan, commercial product	Greenhouse, pots with substrate	Foliar 2, 4, and 6 g L^−1^	Reduction of % postharvest decay, fruit firmness, citric acid.	[100]
Chitosan, commercial product	Greenhouse, pots with substrate	Foliar 1, 2, and 3 g L^−1^	Plant height, number of leaves, leaf aera, dry biomass, fruit size, weight, and yield.	[101]
Chitosan, commercial product	Open field, soil conditions	2.5 and 5 mL L^−1^	Plant height, number of leaves, leaf area, root dry weight, N, P, K, fruit weight, yield.	[102]
Chitosan, commercial product	Open field, soil conditions	Foliar 15 g L^−1^	Fruit firmness, anthocyanin concentration, phenolics and antioxidant capacity.	[103]

**Table 5:** Positive effects of beneficial element applications on some growth or quality variables of strawberry crop.

Product	Experimental Conditions	Forms and Levels of Application	Variables That Increase	Reference
Silicon				
K_2_SiO_3_	Greenhouse, pots with substrate	Drench 1000 and 1500 mg L^−1^	Shoot dry weight, leaf area, root volume, relative water content.	[112]
K_2_SiO_3_	Greenhouse, pots with substrate	Drench 1000 and 1500 mg L^−1^	Plant biomass, fruit number, TSS, TA, antioxidant activity.	[113]
K_2_SiO_3_	Greenhouse, pots with substrate	Drench and Foliar 75 mg L^−1^	General vegetative growth, chlorophyll, stomatal conductance, soluble sugars, CAT, APX, POD, SOD, anthocyanins.	[114]
Si(OH)_4_	Greenhouse, pots with substrate	Drench 1 and 2 mM	Leaf number, leaf area, dry weight, photosynthetic rate, stomatal conductance.	[115]
Si chelate	In vitro	Growing media 2.5, 5, and 10 mg L^−1^	Number and length of shoots, CAT, SOD.	[116]
K_2_SiO_3_	Shade house, pots with substrate	Drench and Foliar 5, 10, and 15 mM	Shoot and root dry weight, chlorophyll, number of flowers and fruits, yield, fruit firmness.	[117]
Si, commercial formulation	Greenhouse, pots with substrate	Foliar 0.3 mL L^−1^	Zn and Si concentration, weight of fruit, yield.	[51]
Na_2_SiO_3_	Greenhouse, soil conditions	Foliar 3 and 6 mM	SOD, phenolics, flavonoids, anthocyanins.	[118]
SiO_2_	Open field, soil conditions	Foliar 5, 10, and 15 mg L^−1^	Fruit firmness and anthocyanins.	[119]
Na_2_SiO_3_	Greenhouse, soilless system	Drench 50 and 100 mg L^−1^	Flavonoids and Si concentration.	[120]
SiO_4_H_4_	Open field, pots with substrate	Drench and Foliar 1.5 mM	Leaf area, SPAD, fruit size and weight, fructose concentration.	[121]
K_2_SiO_3_	Greenhouse, pots with substrate	Drench and Foliar 75 mg L^−1^	Leaf size, fresh and dry weight of shoot, Si concentration, chlorophyll fluorescence.	[122]
Na_2_SiO_3_	Greenhouse, pots with substrate	Drench 3 mM	Shoot and root dry weight, net photosynthesis, relative water content, protein, phenolics.	[123]
K_2_SiO_3_ Na_2_SiO_3_ CaSiO_3_	Greenhouse, pots with substrate	Drench and Foliar 35 and 70 mg L^−1^	CAT, SOD and POD activity.	[124]
Na_2_SiO_3_	Greenhouse, pots with substrate	Drench 3 mM	Shoot and root dry weight, Si, Zn, soluble sugars, soluble proteins, PAL, phenolics.	[125]
Na_2_SiO_3_	Greenhouse, pots with substrate	Drench 3 mM	Shoot and root biomass, net photosynthesis, stomatal conductance, water efficiency use, CAT, SOD, POD.	[126]
K_2_SiO_3_	Shade house, pots with substrate	5, 10, and 15 mM	Root dry weight, chlorophyll fluorescence, net photosynthesis, water efficiency use.	[127]
Selenium				
Na_2_SeO_4_	Greenhouse, soilless system	Nutrient solution 10 and 100 µM	Shoot fresh weight, leaf area, K, Ca, Mg in roots, TSS, fructose, sucrose.	[128]
Na_2_SeO_3_	Greenhouse, pots with soil	Foliar 2.5, 5, and 10 mg L^−1^	Net photosynthesis, stomatal conductance, chlorophyll, SOD, CAT, POD.	[129]
Se NS	Growth chamber, pots with soil	Mix with soil 40 mg kg^−1^ soil	Fruit weight, Se concentration.	[130]
Na_2_SeO_3_	Growth chamber, pots with soil	Foliar 10, 30, and 60 mg L^−1^	Number of fruits, yield, Vit. C, APX.	[131]
Na_2_SeO_3_	Greenhouse, pots with substrate	Drench 2 and 4 mg L^−1^	Fresh and dry weight of crown, K, Ca, Mg, Zn, Se.	[132]
Na_2_SeO_4_	Greenhouse, pots with substrate	Drench 1, 5, and 10 mg L^−1^	Plant biomass, phenolics, flavonoids, antioxidant capacity.	[133]
Iodine				
KIO_3_	Greenhouse, pots with substrate	Drench 1, 2.5, and 7.5 mg L^−1^	Fruit I concentration.	[134]
KI		Foliar 0.25, 0.75, and 1.5 mg L^−1^		
I-based commercial product	Greenhouse, pots with soil	Foliar 0.5 mL L^−1^	Phenolics, APX, CAT, K, I concentration.	[135]
KIO_3_		Foliar 100 µM	Fruit firmness, Vit. C, I concentration.	
KI	Greenhouse, soilless system	Nutrient solution 0.25, 0.5, 1, 2.5, 5 mg L^−1^	Vit. C, soluble sugars, I concentration.	[136]
KIO_3_		Nutrient solution 0.25, 0.5, 1, 2.5, 5 mg L^−1^		
Titanium				
Ti, commercial product	Greenhouse, soil conditions	Soil mix 0.05%	Number of root tips, root dry weight.	[137]
TiO_2_	Greenhouse, soil conditions	Foliar 50, 100, and 150 mg L^−1^	Chlorophylls, yield, glucose, oxalic, malic, and citric acid.	[138]
Ti, commercial product	Open field, soil conditions	Foliar 0.02%	Phenolics, Vit. C, antioxidant capacity, anthocyanins.	[139]

**Table 6:** Positive effects of NM applications on some growth or quality variables of strawberry crop.

Material/Form/Size	Experimental Conditions	Forms and Levels of Application	Variables That Increase	Reference
Se-NPs/spherical/10–45 nm	Greenhouse, pots with substrate	Foliar 10 and 20 mg L^−1^	Root and shot dry weight, number and weight of fruits, yield, chlorophyll concentrations, POD, SOD.	[145]
ZnO NPs 25–50 nm	Open field, soil conditions	Foliar 7.5 × 10^−3^ M	Number of flowers.	[146]
ZnO NPs <100 nm	Open field, soil conditions	Foliar 200, 400, and 600 μg g^−1^	Plant height, number of leaves, leaf area, number of runners, fruit size and yield.	[147]
ZnO NPs NS	Open field, soil conditions	Foliar 50, 100, and 150 mg L^−1^	Plant height, number of leaves, number of fruits and yield.	[148]
Zn NPs NS	Greenhouse, soil conditions	Foliar 10 and 20 mg L^−1^	Number, weight, and fruit yield.	[149]
CeO_2_ NPs 2–50 nm	Greenhouse, soil conditions	Drench 300, 600, 1000, and 2000 mg L^−1^	Shoot and root biomass, root surface area, SPAD.	[150]
CeO_2_ NPs 2–50 nm	Greenhouse, soil conditions	6, 20, 41, 70, and 115 mg L^−1^	Phenolics, Vit. C, soluble protein, IAA, number of fruits.	[151]
Fe NPs NS	In vitro	Growing medium 0.8 mg L^−1^	Shoot length, root dry weight, relative water content.	[152]
Fe NPs NS	In vitro	Growing medium 0.8 mg L^−1^	Branch number, root length, plant weight.	[153]
FeO NPs NS	Open field, soil conditions	Foliar 50, 100, and 150 mg L^−1^	Plant height, number of leaves, number of fruits and yield.	[148]
Fe NPs NS	Greenhouse, soil conditions	Foliar 20 and 40 mg L^−1^	Number, weight, and fruit yield.	[149]
Ag NPs <20 nm	In vitro	Growing medium 0.2, 0.4, 0.6, 0.8, and 1 mg L^−1^	Number and height of shoots, fresh and dry weight, chlorophyll concentration, number and length of roots.	[154]
Se-NPs/10–45 nm	Greenhouse, soil conditions	Foliar 10 and 100 μM	CAT, catechin, caffeic acid, coumaric acid, salicylic acid.	[155]
Se NPs 10–45 nm	Greenhouse, pots with soil	Foliar 25 mg L^−1^	Root fresh weight, chlorophyll, GPX, number of leaves, water efficiency use.	[156]
Ca_5_(PO_4_)_3_(OH) NPs 20–40 nm	Open field, soil conditions	Foliar 15, 30, 60, and 120 mg L^−1^	Fruit postharvest life, firmness, Vit. C.	[157]
SiO_2_ NPs 20–30 nm	Greenhouse, soil conditions	Mix with soil 0.75 and 1.5 g kg^−1^	Root fresh weight, Vit. C, quercetin, proline, PAL, Ca concentration.	[158]
SiO_2_ NPs 20–30 nm	Greenhouse, pots with soil	Foliar 125 mg L^−1^	Number of flowers, anthocyanins, phenolics.	[156]
SiO_2_ NPs NS	Greenhouse, pots with substrate	Drench 50 and 100 mg L^−1^	Shoot and root biomass, chlorophylls, fruit yield.	[159]
SiO_2_ NPs NS	Greenhouse, pots with substrate	Drench 2 mM	Resistance to salt stress through improve membrane stability and decrease H_2_O_2_.	[160]
SiO_2_ NPs 30–35 nm	Shade house, pots with substrate	Drench and Foliar 5, 10, and 15 mM	Shoot and root dry weight, chlorophyll, number of flowers and fruits, yield, fruit firmness.	[117]
Nanozeolite NS	Open field, soil conditions	Mix with soil 5 g bed^−1^	Length of plant, number of leaves, number and weight of fruit and yield.	[161]
Se/SiO_2_ NPs 50–80 nm	Greenhouse, pots with soil	Foliar 50 and 100 mg L^−1^	Shot and root biomass, chlorophyll, CAT, APX, GPX, SOD, fruit size and yield.	[156]
Zn/Fe/Cu NPs NS	Open field, soil conditions	Mix with soil + Foliar 5 mg plant^−1^ + 100 mg L^−1^	Length of plant, number of leaves, Chlorophyll, Vit A, number and weight of fruits, yield.	[161]
ZnO-chitosan 50 nm	Greenhouse, soil conditions	Foliar 400, 800, and 1200 mg L^−1^	Number of leaves, number of fruits, chlorophylls, N, Mg, Mn.	[162]

**Table 7:** Beneficial effects of compost applications on some growth or quality variables of strawberry crop.

Origin of Compost	Experimental Conditions	Forms and Levels of Application	Variables That Increase	Reference
Agricultural waste	Greenhouse Soil conditions	Mix with soil 50% soil–50% compost	Plant dry weight, chlorophyll, fruit weight, TSS, fructose, glucose, sucrose, malic acid, citric acid, yield.	[168]
Chicken manure	High tunnel Soil conditions	Mix with soil 66 g plant^−1^	Plant dry matter, fruit firmness, TSS.	[169]
Vermicompost Chicken manure Cattle manure	Open field Soil conditions	Mix with soil 250 kg ha^−1^	Fruit weight, firmness, yield, TSS, total sugars, Vit. C, N, P, K, Ca, Fe, Zn, Mn, Cu.	[170]
Poultry manure	Greenhouse, pots with soil	0.10 g kg^−1^ soil	Indole Acetic Acid (IAA), Isopentenyl adenosine (iPA).	[66]
Ruminant manure	Open field Soil conditions	150 kg ha^−1^	Fruit yield.	[171]
Cattle manure (compost tea)	Open field Soil conditions	Foliar 8:1 compost:water 1.3 L m^−2^	Fruit yield, resistance to *Botrytis cinerea*.	[172]
Vermicompost	Greenhouse Pots with soil	Mix with soil 200 g kg^−1^ soil	Leaf fresh weight, leaf area, root length.	[173]
Farmyard manure	Open field Soil conditions	Mix with soil 12.5 kg m^−2^	Fruit dry weight, firmness, and yield.	[174]
Chicken manure	Greenhouse Soil conditions	Mix with soil 6 and 12 ton ha^−1^	Plant height, stem thick, fruit yield.	[175]
Mixture of rose oil processing wastes, separated dairy manure, poultry manure, and wheat straws	Greenhouse Pots with substrate	Mix with substrate 12.5, 25, and 50% of total substrate	Number of leaves, number of roots, root length, stem thickness, K, Zn.	[176]
Compost NS	Greenhouse Pots with soil	50% soil and 50% compost 100% compost	Vit. C, GSH, phenolics, anthocyanins.	[168]
Wastes of taif rose petals and red tea leaves	Greenhouse, pots with soil	Mix with soil 1.5 g kg^−1^ soil	Root fresh and dry weight, leaf area.	[177]
Vermicompost from food and paper wastes	High tunnel, soil conditions	Mix with soil 5 and 10 ton ha^−1^	Number of runners and flowers, fruit yield.	[178]
Vermicompost	Greenhouse, pots with soil	50% soil and 50% vermicompost	Plant height, leaf area, number of leaves, plant biomass, fruit weight and yield.	[179]
Vermicompost from cow dung and vegetable waste	Open field, soil conditions	Foliar 2 mL L^−1^	Leaf area, plant biomass, fruit weight, firmness, TSS, yield.	[180]
Vermicompost Mushroom compost Farmyard manure	Open field, soil conditions	170 kg ha^−1^	Number of flowers, yield.	[181]
Farmyard manure Vermicompost	Open field, soil conditions	30 and 80 ton ha^−1^	Plant height, number of leaves, leaf area, number of runners, number, size, and yield of fruits, TSS, Vit. C, phenolics.	[182]

**Table 8:** Favorable effects of H_2_O_2_ and gasotransmitters on some growth or quality variables of strawberry crop.

Product	Experimental Conditions	Forms and Levels of Application	Variables That Increase	Reference
H_2_O_2_				
H_2_O_2_	Greenhouse Hydroponic system (NFT)	Root dipping 1 M	Plant height, root length, leaf number, leaf area, number of adventious roots, plant biomass.	[196]
NO				
Sodium nitroprusside (SNP) as NO source	Greenhouse, pots with substrate	Foliar 50 and 75 μM	Phenolics, SOD, CAT, APX, POD.	[197]
Sodium nitroprusside (SNP) as NO source	Greenhouse, pots with substrate	Foliar 50 and 75 μM	Plant biomass, N, P, K, Ca, Mg, Fe, Zn, Mn, Cu.	[198]
Sodium nitroprusside (SNP) as NO source	Greenhouse, pots with substrate	Foliar 0.1 mM	Shoot biomass, chlorophyll, Fe, CAT, POD.	[199]
Sodium nitroprusside (SNP) as NO source	Greenhouse, pots with substrate	Foliar 75 μM	Vit. C, anthocyanins, phenolics.	[200]
Sodium nitroprusside (SNP) as NO source	Greenhouse, pots with substrate	Foliar 50 and 100 μM	SOD, CAT, APX, GPX, Vit. C, GSH.	[201]
Sodium nitroprusside (SNP) as NO source	Greenhouse, pots with substrate	Foliar 50 and 75 μM	Shoot and root dry weight, leaf area, chlorophyll, number of flowers, fruit size and weight, Vit. C, anthocyanins, phenolics.	[202]
H_2_S				
NaHS as H_2_S source	Greenhouse, pots with substrate	Foliar 0.2 mM	Plant biomass, chlorophyll, SOD, CAT, POD, Zn, Ca, Mg.	[203]
NaHS as H_2_S source	Greenhouse, pots with substrate	Root dipping 100 μΜ	Vit. C, GSH, DHA, heat shock proteins and overexpression of aquaporin-related genes.	[204]
NaHS as H_2_S source	Greenhouse, pots with substrate	Root dipping 0.125, 0.250, 1.250, 2.500, 12.500, 25.000, and 37.500 mM	Length and dry weight of roots, soluble sugars, SOD.	[205]
NaHS as H_2_S source	Greenhouse, pots with substrate	0.2 and 0.5 mM	SPAD, chlorophyll fluorescence, fruit yield, SOD, APX, GR.	[206]
NaHS as H_2_S source	Greenhouse, pots with substrate	Root dipping 100 μΜ	Overexpression of genes such as *cAPX*, *CAT*, *MnSOD*, or *GR*, related with ascorbate-glutathione biosynthesis, transcription factor, and salt overly sensitive pathways.	[207]

**Table 9:** Beneficial effects of PGPR and endophytic bacteria applications on some growth or quality variables of strawberry crop.

PGPR Species	Experimental Conditions	Forms and Levels of Application	Variables That Increase	Reference
Plant Growth-Promoting Rhizobacteria (PGPR)				
*Alcaligenes* *faecalis*, *Staphylococcus* *arlettae*, *S. simulans*, *Agrobacterium rubi*, *Pantoea agglomerans*	Greenhouse, soil conditions	Root dipping 10^8^ CFU mL^−1^	Leaf area, number and weight of fruits, total yield.	[213]
*Bacillus cereus*	Growth chamber, pots with substrate	Mix with substrate 10^6^ CFU g^−1^ substrate	Leaf area, number, weight and yield of fruits, sucrose concentration.	[214]
*Pseudomonas florescence*, *Bacillus subtilis*, *Azotobacter chroococcum*	Open field, soil conditions	Root dipping 10^9^ CFU mL^−1^	Plant height, number of leaves, leaf area, number of runners, chlorophylls, root fresh weight, fruit number, size and yield.	[215]
*Bacillus licheniformis*, *B. subtilis*, *B.* sp. *RG1*, *B.* sp. *S1*, *B.* sp. *S2*	Open field, soil conditions	Root dipping + foliar 10^9^ CFU ml^−1^	Plant height, leaf area, number of runners, number of fruits, yield, chlorophyll, photosynthetic rate.	[216]
*Bacillus subtilis*, *B. atrophaeus*, *B. spharicus*, *Staphylococcus kloosii* *Kocuria erythromyxa*	Open field, soil conditions	Root dipping 10^8^ CFU mL^−1^	Shoot and root dry weight, chlorophyll, relative water content, yield, N, P, K, Ca, Mg, Fe, Mn, Zn, Cu.	[217]
*Pseudomonas BA-8*, *Bacillus OSU-142*, *Bacillus M-3*	Open field, soil conditions	Root dipping + foliar 10^9^ CFU ml^−1^	Fruit yield, total sugars.	[218]
*Bacillus megaterium*, *Bacillus* spp., *Paenibacillus polymyxa*, *Bacillus simplex*	Open field, soil conditions	Root dipping 10^9^ CFU mL^−1^	Number and weight of fruits, TSS, Vit. C, yield.	[219]
*Pseudomonas* sp.	Greenhouse, soil conditions	NS	Plant height, fresh-dry weight, number of runners, number of fruits, yield.	[220]
*Azotobacter chroococcum*, *A.* *vinelandi*, *Derxia* sp., *Bacillus megatherium*, *B. lichenformis*, *B. subtilis*	Open field, soil conditions	Drench 20–40 × 10^6^ CFU mL^−1^	TSS, total sugars, TA, yield.	[221]
*Kocuria E43*, *Alcaligenes 637Ca* *Pseudomonas 53/6*	Greenhouse, pots with soil	Root dipping 10^9^ CFU mL^−1^	Fruit number, weight, and yield, SPAD, stomatal conductance, CAT, SOD, APX.	[222]
*Azospirillum brasilense*	Open field, soil conditions	Root dipping 10^9^ CFU mL^−1^	SPAD, photosynthesis, yield, amino acids and organic acids.	[223]
*Pseudomonas BA-8*, *Bacillus OSU-142*, *Bacillus M-3*	Open field, soil conditions	Root dipping 10^9^ CFU mL^−1^	Fruit yield, P, Fe, Zn.	[224]
*B. methylotrophicus*	In vitro	Growing medium 10^4^ CFU	Shoot and root fresh weight, petiole length.	[225]
Commercial formulation of several PGPR	Open field, soil conditions	Root dipping 10^9^ CFU mL^−1^	CAT, POD, SOD, fruit yield.	[226]
*Azotobacter chroococcum*, *Pseudomonas fluorescens*	Open field, soil conditions	Root dipping 3 × 10^7^ CFU mL^−1^	Plant height, number of leaves, leaf area, number of runners, number, size, and yield of fruits, TSS, Vit. C, phenolics	[182]
Endophytic bacteria				
*B. velezensis*	Greenhouse, pots with substrate	Drench 5 × 10^5^ spores plant^−1^	Shoot and root fresh weight, fruit yield.	[227]
*Arthrobacter agilis*, *B. methylotrophicus*	In vitro	Growing medium 100 μL of bacterial suspension	% Seed germination, shoot fresh weight.	[228]
	Greenhouse	Root dipping 100 μL of bacterial suspension	Fruit yield.	
*Azospirillum brasilense*, *Burkholderia cepacian*, *Enterobacter cloacae*	Greenhouse, pots with soil	Root dipping 10^9^ CFU mL^−1^	Root length and dry weight, aerial dry weight.	[229]
*Azospirillum brasilense*	Growth chamber, pots with substrate	Root dipping 10^6^ CFU ml^−1^	Root length and dry weight, shoot dry weight, total sugars of root exudates.	[230]
*B. amyloliquefaciens*, *Paraburkholderia fungorum*	Open field, soil conditions	Root dipping 10^9^ CFU mL^−1^	Root length, fresh and dry weight, shoot dry weight, fruit weight, anthocyanins, carotenoids, flavonoids, phenolics, antioxidant capacity.	[98]

**Table 10:** Positive effects of arbuscular mycorrhizal fungi and Trichoderma applications on some growth or quality variables of strawberry crop.

Fungi Species	Experimental Conditions	Forms and Levels of Application	Variables That Increase	Reference
Arbuscular Mycorrhizal Fungi (AMF)				
*R. intraradices*	Greenhouse, pots with substrate	0.5 g plant^−1^	CO_2_ assimilation, stomatal conductance, relative water content.	[235]
*G. mosseae*, *G. aggregatum*	Greenhouse, pots with substrate	NS	P concentration, free amino acids concentration.	[236]
*G. mosseae*	Greenhouse, pots with soil	1 g plant^−1^	Dry weight of shoots, phenolics, antioxidant activity, SOD.	[237]
*F. mosseae*, *F. geosporus*, *C. claroideum*, *G. microagregatum*, *R. irregularis*	Greenhouse, pots with substrate	20 g plant^−1^	Fruit yield, root length.	[238]
*G. intraradices*	Greenhouse, pots with substrate	2 mL plant^−1^ from solution of 50 g L^−1^	K, Cu, phenolics, anthocyanins, flavonoids.	[239]
*G. intraradices*	Open field, soil conditions	1 g plant^−1^	Root biomass, daughter plants per mother plant.	[240]
*R. clarus*	Greenhouse, pots with substrate	60 g plant^−1^	Shoot and root biomass, relative water content, net photosynthesis.	[126]
*F. mosseae*, *F. geosporus*	Greenhouse, pots with substrate	1:10 inoculated substrate: growing substrate mix	Shoot and root length and fresh weight, SPAD, fruit weight.	[241]
Mix of various *Glomus* species	Greenhouse, pots with substrate	100 mL of mycorrhizal preparation plant^−1^	Anthocyanins concentration.	[242]
*G. fasciculatum*, *G. etunicatum*	Greenhouse, pots with substrate	2.5 g plant^−1^	Shoot dry weight, P and K concentration.	[243]
*G. irregularis*	Greenhouse, pots with substrate	80–100 spores plant^−1^	Length, volume, and dry weight of roots.	[244]
*Cetraspora pellucida*, *Claroideoglomus etunicatum* and mycorrhizal community	Greenhouse, pots with substrate	10 g plant^−1^	Aerial biomass, root length and biomass, anthocyanins, flavonoids, phenolics.	[245]
*Gigaspora* *margarita*	Greenhouse, pots with soil	30 spores plant^−1^	Root biomass, Mg, Mn.	[246]
*G. clarum*			P, Mg, Ca, S, Fe, Cu, Zn.	
*Gigaspora rosea*			N, P, Mg, Ca, S, Fe, Cu, Mn Zn.	
*G. mosseae*, *G. intraradices*	Greenhouse, pots with substrate	20 spores g^−1^ of substrate	SPAD, number of leaves and flowers, number of fruits.	[247]
*G. mosseae*	NS	10% of inoculated substrate	Plant height, leaf area, fresh and dry weight of shoot and roots, chlorophyll.	[248]
AMF NS	Open field, soil conditions	20 g plant^−1^	Plant height, biomass, fruit size, yield.	[180]
*Trichoderma*				
*T. harzianum* *T. virens*	Greenhouse, pots with soil	25 mL plant^−1^ (10^7^ spores mL^−1^)	Root length and dry weight, number of fruits, yield, Vit. C, anthocyanins.	[249]
*T. harzianum* *T. viride*	Open field, soil conditions	Root dipping in fungi preparation (10^6^ spores mL^−1^)	Root biomass, fruit yield.	[250]
*T. citrinoviride*	Greenhouse, pots with substrate	Root dipping in fungi preparation (2 × 10^6^ CFU mL^−1^)	Plant dry weight, PSII efficiency.	[251]
*T. harzianum*	Greenhouse, pots with soil	50 mL plant^−1^ (9.90 × 10^6^ CFU 100 mL^−1^)	Vegetative growth, number of flowers, number, weight, and yield of fruits, TSS, TA, Vit. C.	[252]
*T. viride*	NS	10% of inoculated substrate	Plant height, leaf area, fresh and dry weight of shoot and roots, chlorophyll.	[248]

**Table 11:** Positive effects of UV and visible light supplementation on some growth or quality variables of strawberry crop.

Light Source	Experimental Conditions	Wavelength (nm)/Photosynthetic Photon Flux Density (PPFD) (µmol m^−2^ s^−1^)	Variables That Increase	Reference
LED	Greenhouse, pots with substrate	450–550/400	Photosynthetic rate, leaf area, leaf dry weight, fruit number, weight, yield, TSS and firmness.	[264]
Fluorescent lamp (FL)		405–610/NS	Photosynthetic rate, leaf area, leaf dry weight.	
Blue LED	Greenhouse, pots with substrate	447/335	Leaf area, number of leaves, number of flowers, N, K, Ca, Fe, Mn, and Zn concentration.	[257]
Red LED		666/375		
White LED		494/330		
FL		479/275		
FL+UV		480/314		
Red:Blue LED (8:2)	Greenhouse, pots with substrate	445–659/106–117	Number of leaves, crown diameter, plant dry weight, number of flowers, number, and weight of fruits, TSS, Vit. C.	[65]
Red:Blue LED (5:5)		445–659/107–125	Crown diameter, plant dry weight, TSS of fruits.	
Red:Blue LED (2:8)		445–659/105–121	Crown diameter, plant dry weight, K concentration.	
Blue LED	Greenhouse, pots with substrate	448/75	Fruit yield, glucose concentration.	[265]
Red LED		661/75	Sucrose, citric acid, malic acid concentration.	
Blue + Red LED		634/75	Fruit yield, fructose, glucose	
Red LED	In vitro	660/45	Plant height, number of leaves, root length.	[266]
Blue LED	Greenhouse, pots with substrate	470/190	Days to anthesis, fruit yield.	[267]
Light with various color temperatures (3000, 4000, 5000, and 6500 K)	Growth chamber, pots with substrate	NS	Leaf number and size, crown diameter, dry weight of plant, SPAD.	[268]
Red, Blue and Red:Blue LED	Greenhouse, pots with substrate	450–730/190	Fruit anthocyanins and proanthocyanins.	[269]
LED NS	Greenhouse, pots with substrate	450–550/400	Less days to flowering, number of flowers, dry biomass of plant, number, weight, and yield of fruits, TSS, firmness.	[270]
Red LED	Greenhouse, pots with substrate	660/200	Leaf fresh weight, fruit number and size.	[271]
Blue/Red		460–660/200	Leaf fresh weight, leaf area, SPAD, fruit number and size, TSS.	
White–Yellow		400–700/200	Leaf fresh weight, crown fresh weight, SPAD, fruit number and size.	
Red LED	Greenhouse, pots with substrate	660/200	CO_2_ assimilation rate, water use efficiency, stomatal conductance, transpiration.	[272]
Blue/Red		460–660/200		
White–Yellow		400–700/200		

## Data Availability

Not applicable.
